# GDF5 Regulates TGFß-Dependent Angiogenesis in Breast Carcinoma MCF-7 Cells: In Vitro and In Vivo Control by Anti-TGFß Peptides

**DOI:** 10.1371/journal.pone.0050342

**Published:** 2012-11-30

**Authors:** Francesca Margheri, Nicola Schiavone, Laura Papucci, Lucia Magnelli, Simona Serratì, Anastasia Chillà, Anna Laurenzana, Francesca Bianchini, Lido Calorini, Eugenio Torre, Javier Dotor, Esperanza Feijoo, Gabriella Fibbi, Mario Del Rosso

**Affiliations:** 1 Department of Experimental Pathology and Oncology, University of Florence, Florence, Italy; 2 Istituto Toscano Tumori, Florence, Italy; 3 Department of Oncohematology, Istituto Tumori Giovanni Paolo II, Bari, Italy; 4 DIGNA Biotech, Pamplona, Spain; University of Bari Medical School, Italy

## Abstract

**Background:**

TGFß overproduction in cancer cells is one of the main characteristics of late tumor progression being implicated in metastasis, tumor growth, angiogenesis and immune response. We investigated the therapeutic efficacy of anti-TGFß peptides in the control of angiogenesis elicited by conditional over-expression of TGFß.

**Methods:**

We have inserted in human MCF7 mammary-cancer cells a mutated TGFß gene in a tetracycline-repressible vector to obtain conditional expression of mature TGFß upon transient transfection, evaluated the signaling pathways involved in TGFß-dependent endothelial cells activation and the efficacy of anti-TGFß peptides in the control of MCF7-TGFß-dependent angiogenesis.

**Results:**

TGFß over-expression induced in MCF7 several markers of the epithelial-to-mesenchymal transition. Conditioned-medium of TGFß-transfected MCF7 stimulated angiogenesis in vivo and in vitro by subsequent activation of SMAD2/3 and SMAD1/5 signaling in endothelial cells, as well as SMAD4 nuclear translocation, resulting in over-expression of the pro-angiogenic growth and differentiation factor-5 (GDF5). Inhibition or silencing of GDF5 in TGFß-stimulated EC resulted in impairment of GDF5 expression and of TGFß-dependent urokinase-plasminogen activator receptor (uPAR) overproduction, leading to angiogenesis impairment. Two different TGFß antagonist peptides inhibited all the angiogenesis-related properties elicited in EC by exogenous and conditionally-expressed TGFß in vivo and in vitro, including SMAD1/5 phosphorylation, SMAD4 nuclear translocation, GDF5 and uPAR overexpression. Antagonist peptides and anti-GDF5 antibodies efficiently inhibited in vitro and in vivo angiogenesis.

**Conclusions:**

TGFß produced by breast cancer cells induces in endothelial cells expression of GDF5, which in turn stimulates angiogenesis both in vitro and in vivo. Angiogenesis activation is rapid and the involved mechanism is totally opposed to the old and controversial dogma about the AKL5/ALK1 balance. The GDF-dependent pro-angiogenic effects of TGFß are controlled by anti-TGFß peptides and anti-GDF5 antibodies, providing a basis to develop targeted clinical studies.

## Introduction

Transforming growth factor beta-1 (TGFß), a multifunctional cytokine initially identified as a “transforming” growth factor by its property to induce malignant behaviour of normal fibroblasts in culture [1], was later shown to promote profound growth-suppressive effects on many cells and was therefore taken into consideration as a candidate tumor suppressor gene [2], [3]. However, it was soon discovered that metastasis of many different types of tumors actually requires TGFß activity and that, in the context of advanced disease, it has prooncogenic effects [4]. The current understanding of the role of TGFß in cancer indicates that TGFß suppresses the progression of early lesions, but later this effect is lost and cancer cells themselves produce TGFß that promotes the metastatic process [5]. TGFß inhibits mammary tumorigenesis by directly inducing mammary epithelial cells to stop cell cycle, to undergo apoptosis and to release a complex array of cytokines, growth factors and extracellular matrix proteins that maintain mammary tissue homeostasis [6], [7]. The events related with the acquisition of malignancy-related properties by TGFß identify a critical phase of tumor progression, which has been named “TGFß-switch” [5], [8], connoted by loss of TGFß-dependent growth inhibition, apoptosis and genomic stability, and by increased expression/activation of TGFß (reviewed in refs. [8], [9]) which profoundly affects tumor cells and their microenvironment. Accordingly, in breast cancer higher levels of TGFß are often detected in tumors when compared to corresponding normal mammary gland, and the difference appears even higher in the most advanced stages of mammary tumor progression [10], producing a micro-environment that promotes tumor growth, epithelial-mesenchymal transition (EMT), survival and invasion/motility of cancer cells, modulation of a set of pro-metastatic genes that govern the pattern of osteoclast activation in the sites of bone colonization of cancer cells [11], immuno-suppression and angiogenesis [12], [13].

Also angiogenesis regulation reflects the opposite activities of TGFß. The effect of TGFß on angiogenesis has been shown to be context-dependent [14], [15]: at low concentrations TGFß promotes endothelial cells (EC) proliferation and migration, whereas at high concentration it has the opposite effect [14]–[17]. In bovine capillary EC, TGFß signaling converts the VEGF/VEGF receptor-2 (flk-1)-mediated activation of p38^MAPK^ into a pro-apoptotic signal [18], while protracted treatment of the same EC with TGFß results into EC remodeling and induction of cord-like structures [19]. TGFß has been shown to induce expression of selected members of the VEGF family in EC [20] and carcinoma cells [21]. Knockouts for TGFß and its receptors show defects in angiogenesis, and often die *in utero* [22]. In conclusion, TGFß acts either as inhibitor or enhancer of neo-vascularization in many tumors, including breast cancer [6].

The opposing effects of TGFß on angiogenesis have been explained by the differential involvement of two distinct signalling cascades. TGFß can bind to and signal through two different type-I receptors in EC, the ubiquitous TGFBR1 activin-like receptor kinase 5 (ALK5) and the EC-restricted ACVRL1 (ALK1), resulting in activation of SMAD2/3 and SMAD1/5, respectively [14], [15], [23]. Through ALK5 TGFß determines the terminal maturation phase of angiogenesis [24]–[26], while ALK1 stimulates EC proliferation and migration [27]. Therefore, while the ubiquitous ALK5 has a role in vascular maturation, the EC-restricted ALK1 promotes neo-angiogenesis. It was thus suggested that the balance between TGFß/ALK1 versus TGFß/ALK5 will determine the pro- or anti-angiogenic effects of TGFß. However, gain-of-function studies have shown that ALK1 signaling inhibits proliferation and migration of human microvascular EC, implying an involvenment of ALK1 in the resolution phase of angiogenesis [28]. Further, similar to TGFß, another member of the TGFß family, the bone morphogenetic protein 9 (BMP9), is a high-affinity ligand for ALK1 in EC and exhibits both anti-angiogenic [29], [30] and pro-angiogenic effects on Matrigel plug vascularization and in a xenograft model of pancreatic cancer [31]. Nevertheless, TGFß and BMP9 have been shown to synergize in improving EC response to angiogenic FGF2 and VEGF stimulation [32]. Within this complex scenario, the effects of TGFß on tumor angiogenesis seem to be referred to direct stimulation of the ALK1-SMAD1/5 signaling in EC of the tumor microenvironment [12], [33], or to promotion of VEGF expression [20], [34]. We have recently reported that TGFß at low concentrations (1 ng/ml) exerts an angiogenic activity on microvascular EC in a Matrigel model system, both *in vitro* and *in vivo*, by up-regulating urokinase-type plasminogen activator (uPA) receptor (uPAR) expression [17].

Because of all these considerations, in the present work we have used anti-TGFß peptides to blunt the pro-angiogenic activity of the MCF-7 breast cancer cell line transfected with a TGFß gene under the control of a tetracycline repressed CMV promoter (Tet-Off system). We have observed that TGFß promotes angiogenesis by inducing EC overproduction of the pro-angiogenic growth and differentiation Factor-5 (GDF5) (also called Bone Morphogenetic Protein-14, BMP14), which controls uPAR up-regulation in EC. Two peptides, previously used in another study of our laboratory [17], inhibited breast tumor TGFß-dependent angiogenesis *in vitro* and *in vivo* by inhibition of TGFß signaling and of the subsequent TGFß-dependent GDF5 overproduction in EC.

## Materials and Methods

### Ethics Statement

The local Institutional Animal Care and Use Committee of the Medicine Faculty of Florence (Ospedale di Careggi) and the Italian Ministry of Health (Ministerial Decree n 21/2010, released on January 28, 2010) approved the experimental protocols described in the study.

### Cell Lines

Human dermal microvascular endothelial cells (MVECs) were purchased from Lonza Ltd. Cells were maintained in complete endothelial cell growth medium (ECGM), as described [35], [36]. MVECs were used between the third and seventh passage in culture. The breast cancer MCF7 Tet-Off Avanced cell line (BD Clontech, Inc.) was grown in DMEM supplemented with 10% fetal bovine serum, 1% penicillin-streptomycin, and 100 µg/ml Geneticin (G418; all supplied by Life Technologies, Inc.).

### Antagonist TGFß peptides

We have used two TGFß antagonist peptides developed by Digna Biotech (Pamplona, Spain), one derived from its type III receptor [37]: peptide p144 (TSLDASIIWAMMQN, 1580.86 Da); the other one derived from phage display library technology [38]: peptide p17 (KRIWFIPRSSWYERA, 1995.6 Da). While p17 is water soluble, p144 is partially hydrophobic. The final concentration of 100 µg/ml antagonist peptides was chosen on the basis of previous results [17], [37], [38].

### TGFß construct

The construct pTRETGF-beta1 containg the TGFß1 coding sequence mutated at codon 223 (Cys→Ser) and 225 (Cys→Ser) in pTRE2pur plasmid (Clontech) was produced in collaboration with PRIMM srl, Milan, Italy. Briefly the TGFß1 sequence was synthesized with Right Gene® technology using the optimal codon usage for *Homo sapiens*. The synthetic gene, producing an activated TGFß1, was inserted into the BamHI and NotI restriction site of the pTRE2pur vector.

### Transient Transfection

The cDNA-containing vector and the empty plasmid were transiently transfected into MCF7 Tet-Off Advanced cells using Lipofectamine Tranfection Reagents (Invitrogen Co.), according to the manufacturer's protocol. Briefly, 3.5×10^5^ cells/well were plated in six multiwell plates 24 hrs before transfection. 24 hrs after transfection, complete medium was replaced, supplemented or not with 1 µg/ml of doxycycline.

### Measurement of TGFß levels in conditioned-medium from Mock and TGFß transfected cells

Levels of TGFß in Mock and TGFß-transfected MCF7 (MCFM and MCFTß, respectively) media were measured by a commercial ELISA kit (eBioscence; Austria) according to the manufacturer protocol. Determinations were done in quadruplicate and were expressed as pg/ml.

### Preparation of Conditioned-Media

Multi-well plates (6 wells) of MCFM and MCFTß were washed with PBS and maintained 72 hours in 2 ml culture medium supplemented with 2% fetal bovine serum, in the presence or in the absence of doxycycline. The conditioned-medium (CM) was then subjected to TGFß determination and properly diluted for *in vitro* capillary morphogenesis and Western blot analysis.

### Invasion assay

The Boyden chamber assay, where upper and lower wells were separated by a porous membrane coated with Matrigel, was used to evaluate cell invasion as described [35]. A total of 10^5^ MCFM or MCFTß cells were placed in the upper chamber in culture medium added with 2% fetal calf serum and migration was allowed to occur for 24 hours at 37°C in 5% CO2. Mobilization was measured by counting the number of cells moving across the filter. Each point was performed in triplicate. Migration was expressed as the number of migrated cells ± SD.

### Cell Viability Assay

The viability of MCFM and MCFTß cells was determined by a cell proliferation assay using WST-1 reagent (Roche). WST-1 is a water-soluble sulfonated tetrazolium salt that is cleaved by cellular succinate-dehydrogenases in living cells, yielding dark blue formazan. Damaged or dead cells exhibit reduced or no dehydrogenase activity. Briefly, Tet-Off MCFM and MCFTß cells 24 hours after transfection were plated onto a 96-multiwell plate in quadruple. After 24, 48 and 72 hours WST-1 solution/culture medium (5 mmol/l, 1∶9) was added to each well. Following 2-hours incubation at 37°C, absorbance at 450 nm (reference at 630 nm) was measured by a Multiskan JX microplate reader. Percentage of cell viability was calculated based on the absorbance measured relative to that of the untreated control cells maintained in culture medium alone.

### *In vitro* capillary morphogenesis assay

Capillary morphogenesis experiments on Matrigel were performed as previoiously described [35] by plating 60×10^3^ MVECs/well. Capillary morphogenesis was evaluated after 6 and 18 hours using an inverted microscope (Leitz DM-IRB) equipped with CCD optics and a digital analysis system (Image J 1.44 software, NIH, Bethesda, MD). Some experiments were performed in the presence of anti-GDF5 or anti-BMP7 antibodies (GeneTex), as well as with irrelevant IgGs. Results were quantified by measuring the extent of field occupancy of capillary projections (expressed as the mean percentage ±SD, relative to control values set at 100%). Six-to-nine photographic fields from 3 plates were scanned for each point. Some experiments were performed in the presence of 5 µg/ml anti-GDF5 or irrelevant mouse IgG.

### *In vivo* Matrigel Sponge Assay

An amount of 2.5×10^6^ MCFM or MCFTß, was injected subcutaneously in 6–8 weeks old SCID mice (Charles River). Cell suspensions were added to un-polymerized Matrigel at 4°C containing 50 U/ml heparin at a final volume of 0.6 ml. The Matrigel suspension was injected subcutaneously into the flanks of 4 mice for each condition (two plugs/mice) using a cold syringe. At body temperature, the Matrigel polymerizes to a solid gel, which becomes vascularized within 4 days in response to pro-angiogenic substances. Pellets were removed and photographed. One pellet from each mouse was formalin-fixed for immunohistochemistry. The pellet was minced, and diluted in water to measure the haemoglobin content with a Drabkin reagent kit (Sigma). Similar experiments were performed also in the presence of the TGFß antagonist peptides p17, p144 or of a scramble peptide (Digna Biotech, Pamplona, Spain) [17] added to the Matrigel mix at final concentration of 100 µg/ml. In other experiments p17 was administered daily to mice by an intraperitoneal injection of 100 µl of a 1 mg/ml solution, until a total amount of 500 µg/animal was reached. Further, sponge vascularization was evaluated in C57/BL6 male mice injected with Matrigel containing CM of MCFTß cells obtained after doxycycline subtraction, with or without GDF5-siRNA or not targeting-siRNA, combined with DharmaFECT.

### Immunohistochemistry to evaluate matrigel sponge vessel density

After fixation in formalin, the sponge specimens were embedded in paraffin. Serial sections were stained with H&E or with a primary rabbit polyclonal anti-CD31 IgG (ab28364, abcam United Kingdom, dilution 1∶50), following routine immunohistological methods: HRP-conjugated secondary IgG (Sigma-Aldrich, 1∶200, 30 min) was revealed with 3,3′-Diaminobenzidine as chromogen (5 minutes), using hematoxyline as counterstain. Counting of CD31-positive vessel structures was performed with a 400-time magnification using a squared reference grid. Vessel counting was performed in ten to fifteen fields. Statistical significance of the different vessel counts was determined using Student's *t*-test where *p*≤0.05 was considered statistically significant.

### Western Blot Analysis

Conditioned-media of MCF7 cells (100 µl) and aliquots of MCF7 and MVECs lysates (50 µg) were obtained, processed, and blotted as described [35], [39]. The primary antibodies were: anti-Vimentin (1∶1000) (Sigma-Aldrich), anti-E-cadherin (1∶500), anti-N-cadherin (1∶500) (Santa Cruz), anti-TGFß (1∶250, catalog n. sc-146; Santa Cruz); anti-phospho-SMAD1/5 (p-SMAD 1/5) (1∶500, catalog n. 9516), anti-SMAD5 (1∶500, catalog n. 9517), SMAD2/3 (100 µg/ml, 1∶500; catalog n. 3102), and phospho-SMAD2 (Ser 465/467) (p-SMAD 2) (100 µg/ml, 1∶500) (catalog n. 3108) (all from Cell Signaling Technology, Inc.), anti-GDF5 (650 µg/ml, 1∶1000; catalog n. GTX113580; GeneTex) and anti–alpha Tubulin (1∶1000, catalog n. 3931; Sigma-Aldrich). uPAR expression in MVECs was evaluated by anti-uPAR M2 (1 µg/ml, rabbit polyclonal antibody), kindly provided by Dr. D'Alessio (DIBIT, San Raffaele Scientific Institute, Milan, Italy), previously used in our laboratory [39]. Also MCF7 apoptosis was evaluated by Western blot, using a monoclonal anti-caspase-3 (100 µg/ml, 1∶500) (cat. Sc-7272, Santa Cruz). Protein-antibody complexes were revealed by an Odyssey Infrared Imaging System, using IRDye 800 CW goat antimouse IgG (1∶12,000, cat. FE30926220; LI-COR Biosciences) and Alexa Fluor 680 goat antirabbit IgG (1∶12,000, cat. A21076; Invitrogen) as fluorescent secondary antibodies according to the instructions provided by the manufacturers.

### Immunofluorescence confocal microscopy

Immunofluorescence was performed as previously described [39]. Mock and TGFß-MCF7 Tet-Off cells were placed on coverslips in DMEM-10% FCS, fixed in paraformaldehyde and permeabilised according to routine methods. The primary antibodies were: anti-Vimentin (1∶1000) (Sigma-Aldrich), anti-E-cadherin (1∶500), anti-N-cadherin (1∶500) (Santa Cruz). Secondary antibodies were anti-Rabbit IgG (whole molecule)-Cy3 conjugated (1∶1000) (Sigma-Aldrich), anti-Mouse IgG (whole molecule)-FITC conjugated. MVECs were grown on coverslips in ECGM that after 24 h was replaced with CM with or without the TGFß antagonist peptides. After 1 and 2 hours MVECs were fixed and permeablised. The anti-human primary antibody was anti-SMAD4 (1∶200) (catalog n. 3102; Cell Signaling), the secondary antibody was CY3-conjugated anti-mouse IgG (1∶800) (catalog n. C2181, Sigma). For nuclear staining, the samples were incubated with DAPI (2 µg/ml), for 15 min. Coverslips were observed under a Bio-Rad MRC 1024 ES Confocal Laser Scanning microscope (Bio-Rad, Hercules, CA). A single composite image was obtained by superimposition of 20 optical sections (thickness of 1 µm at intervals of 0.8 µm for each sample). Collected images were analyzed by ImageJ software (NIH, Bethesda, MD).

### Quantitative RT-PCR array for TGFß pathways activation

Total RNA was prepared using Nucleospin RNA II (Macherey-Nagel, Germany), agarose gel checked for integrity, and reverse transcribed with iScript cDNA Synthesis Kit (Bio-rad, ) using random primers according to manufacturer's instructions. The StellArray system (LONZA Ltd., Switzerland), a qPCR based assay in 96 well format was used for quantitative profiling of a set of 94 preselected genes related to TGFß pathway. For comparison of the quantitative gene expression profiles of cells under different culture conditions, fold changes above and below 3 of analyzed genes were plotted. Ct values were analyzed by Global Pattern Recognition (GPR) data analysis tool (Bar Harbor Biotechnology, USA) [40].

### siRNA treatment

MVECs were transfected with 40 nM GDF5-siRNA, BMP7-siRNA, or control not-targeting siRNA (Dharmacon) using Lipofectamine 2000. After 48 h treated cells were harvested, plated on a Matrigel layer in 2% FCS and observed for capillary morphogenesis. CMs from GDF5-siRNA and control not-targeting siRNA-treated-MCF7 cells were also used in Matrigel sponge assay angiogenesis. In order to evaluate the effectiveness of GDF5 silencing and its effect on uPAR expression, quantitative PCR was performed with an Applied 7500 Fast apparatus using reagents provided by the manufacturer (SYBR Green Master mix, Applied Biosystems, CA, USA). Total mRNA was extracted and retrotranscribed as described above. A primer pair (Forward: 5′-AGGCAACAGCAGCGTGAAGT-3′; Reverse: 5′-GGTCATCTTGCCCTTTGTCAA-3′) able to amplify a 76 bp fragment from the human GDF5 cDNA, was designed with Primer Express 3.0 software and purchased from Integrated DNA Technologies (Belgium, EU). RNA amount was evaluated using the ΔΔCt method normalizing to 18 s RNA (Forward: 5′-CGGCTACCACATCCAAGGAA-3′ and reverse 5′-GCTGGAATTACCGCGGCT-3′). uPAR primers and cycling conditions were as previously reported [39].

### RT-PCR analysis of ALKs receptors

RT-PCR analysis of ALKs receptors was determined by an internal-based semi-quantitative RT-PCR, using procedures previously described [41]. The primers' sequences, the size of products and cycling profile are reported in Table 1. The reaction products were analyzed by electrophoresis in 1% agarose gel containing ethidium bromide and the amount of each amplification product was determined by densitometer analysis.

**Table 1:** PCR primers and cycling conditions.

Primer	Sequence	Size (bp)	Cycling profile
**ALK1**	5′-ATTACCTGGACATCGGCAAC-3′ (sense)	214	94°C, 1 min; 59°C , 1 min; 72°C, 1 min; 35 cycles total
	5′-TTGGGCACCACATCATAGAA-3′ (antisense)		
**ALK2**	5′-TCAGGAAGTGGCTCTGGTCT-3′ (sense)	180	94°C, 1 min; 59°C , 1 min; 72°C, 1 min; 35 cycles total
	5′-CGTTTCCCTGAACCATGACT-3′ (antisense)		
**ALK3**	5′-CGTTTCCCTGAACCATGACT-3′ (sense)	137	94°C, 1 min; 59°C , 1 min; 72°C, 1 min; 35 cycles total
	5′-AGCCCTACATCATGGCTGAC-3′ (antisense)		
**ALK5**	5′- ATCCCAAACAGATGGCAGAG-3′ (sense)	178	94°C, 1 min; 59°C , 1 min; 72°C, 1 min; 35 cycles total
	5′- GGAGAGTTCAGGCAAAGC TG-3′ (antisense)		
**ALK6**	5′-AAGTTACGCCCCTCATTC-3′ (sense)	244	94°C, 1 min; 59°C , 1 min; 72°C, 1 min; 35 cycles total
	5′- TGATGTCTTTTGCTCTGC-3′ (antisense)		
**GAPDH**	5′-CCACCCATGGCAAATTCCATGGCA-3′ (sense)	598	94°C, 1 min; 56°C , 1 min;72°C, 1 min; 35 cycles total
	5′-TCTAGACGGCAGGTCAGGTCCACC-3′ (antisense)		

### Statistical analysis

Results are expressed as means ± SD. Multiple comparisons were performed by the Student test. Statistical significances were accepted at p<0.05.

## Results

### A mutated TGFß gene inserted in an inducible vector results in conditional expression of mature TGFß upon transient transfection

#### Effects of TGFß overproduction on MCF7 cells

**Figure 1:**
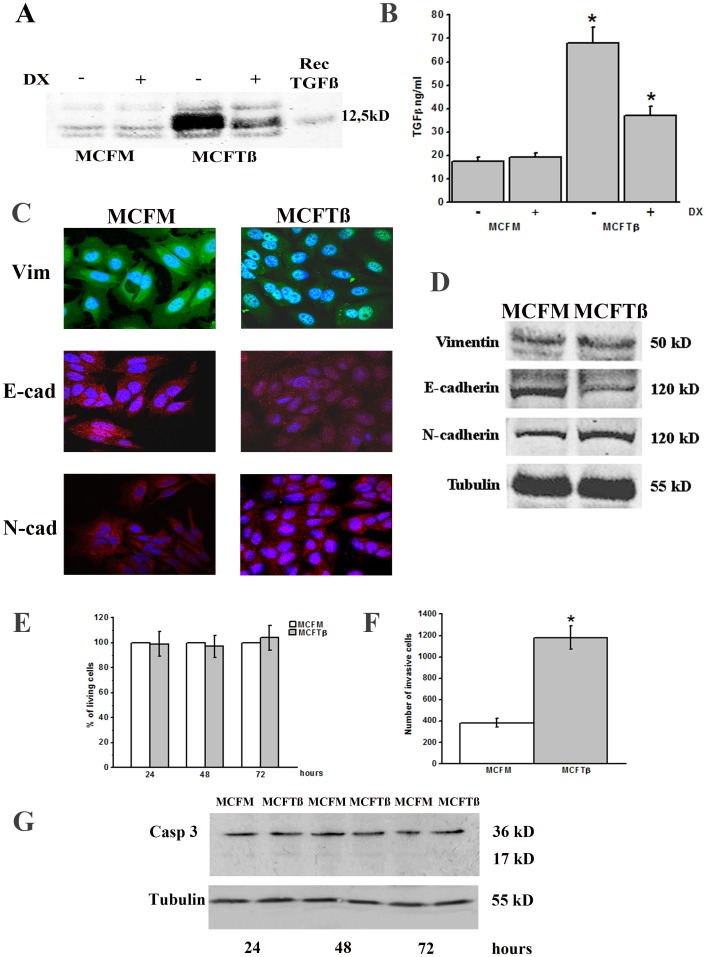
TGFß expression in transfected MCF7 cells. Effects of TGFß overproduction on MCF7 cells. **Panel A**. Western blot of conditioned-media. MCFM: MCF7-Tet-Off transfected with the mock empty vector; MCFTß: MCF7-Tet-Off transfected with the mutated TGFß1-coding sequence. The figure reports a typical experiment out of 4 replicas. On the right, molecular weight expressed in kDa; Dx = Doxycycline. **Panel B**. ELISA kit determination of TGFß concentration in the culture medium of MCF7 in the same conditions as in panel A (4 different experiments performed in triplicate). * p<0.05 (Student t-test), significantly different from mock. **Panel C**. Confocal microscopy showing distribution of vimentin, E-cadherin and N-cadherin in MCFM and MCFTß. Original magnification: ×60. **Panel D**. Western blot of cell lysates. On the right, molecular weight expressed in kDa. Tubulin: loading control. The figures report a typical experiment out of 4 replicas. **Panel E**. Kinetics of % vital cells obtained by using the WST-1 reagent. Data are shown as the mean ±SD obtained in 4 different experiments performed in triplicate in the reported conditions. **Panel F**. MCFM and MCFTß cells Matrigel invasion in the Boyden chamber assay. Data are expressed as percent variations of invasive cells against the untreated control, taken as 100%, ±SD, obtained in 4 different experiments performed in triplicate. * p<0.05 (Student t-test), significantly different from mock. **Panel G**. Western blotting showing caspase activation. On the right, molecular weight expressed in kDa. Tubulin: loading control. The figure reports a typical experiment out of 4 replicas.

In order to obtain a cell model recapitulating the increase of TGFß expression/activation, a mutated TGFß gene (coding for activated TGFß) was cloned under the control of a tetracycline-repressed CMV promoter. Tet-Off MCF-7 cells were transiently transfected with the inducible construct or the empty vector. Twenty-four hours after transfection the medium was replaced with a serum-free medium containing or not doxycyclin. The supernatants were collected after five days and assayed for TGFß by western blot analysis (figure 1, panel A). The western blot analysis revealed a difference of about ten folds in the content of 12,5 kD TGFß monomer in media from MCF7 cells transfected with TGFß (MCFTß) with respect to the media from the mock transfected sample (MCFM), and a difference of more than two folds was observed with respect to the media from doxycycline-treated TGFß expressing cells. The difference in TGFß content between media from TGFß transfected cells treated with doxycycline and media from mock transfected cells was *bona fide* due to residual mature TGFß present inside the cells and to residual transcriptional/translational activity; in fact, Western blot analysis (not shown) demonstrated that TGFß precursor forms returned to the basal level within 72 hours upon doxycycline treatment and, therefore, that residual presence of TGFß in the presence of doxycycline was not related to leakage of bad functioning of the system. Results in agreement with those obtained with Western blot were obtained by ELISA TGFß quantification in MCFM and MCFTß media collected after five days from doxycycline withdrawal. (figure 1, panel B).

Panel C of figure 1 shows that TGFß over-expression induced in MCF7 nuclear translocation of vimentin [42], decrease of cytoplasmic E-cadherin and increase of N-cadherin [43]. Panel D shows the Western blotting of the relevant molecules. Further, cell viability was not decreased (figure 1, panel E) and apoptosis (evaluated by caspase-3 activation, figure 1, panel G) was not increased by TGFß over-expression. MCF7 invasion of Matrigel-coated filters resulted amplified (figure 1, panel F). Taken together, all these features recapitulate several characteristics of an epithelial-to-mesenchymal (EMT) transition.

### Evaluation of the angiogenic effects of TGFß over-expressing cells *in vitro* and *in vivo*

#### Inhibition by anti-TGFß peptides

**Figure 2:**
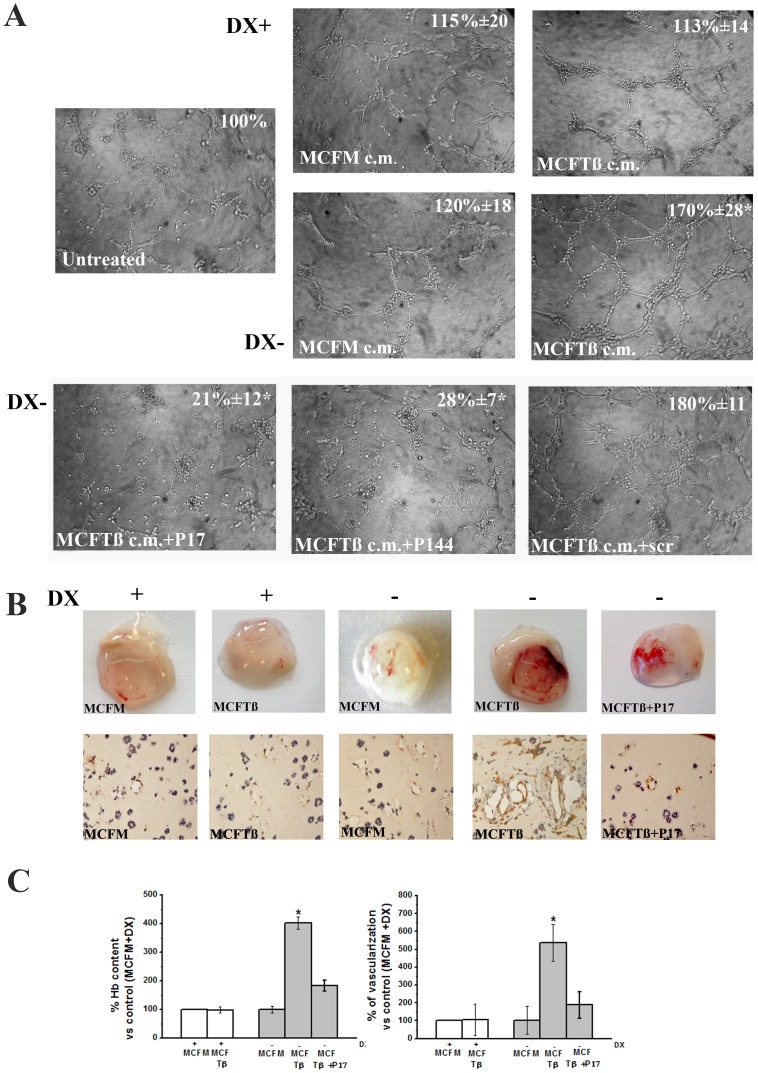
*In vitro* and *in vivo* evaluation of MCF7-TGFß-induced angiogenesis. **Panel A**. MVECs were exposed for 6 hours to CM from MCFTß or MCFM. Numbers on the upper right side indicate the percent field occupancy of capillary plexus (±SD), taking untreated MVECs incubated in their own culture medium as 100%. Since the overall morphology did not substantially differ between 6 and 18 h after MVEC seeding (apart of a better definition of tubule formation by capillary projections), quantification was performed at 6 h after seeding. Pictures show a typical out of 4 different experiments that gave similar results. Dx: Doxycycline. The bottom pictures of panel A show capillary morphogenesis of MVECs exposed to CM of Dx−/MCFTß in the presence of p17 (+P17), p144 (+P144) or of a scrambled oligopeptide (+scr), respectively. * p<0.05 (Student t-test), significantly different from mock. **Panel B**. Upper part: Matrigel sponges, containing 2.5×10^6^ MCFTß or MCFM, were recovered five days post implantation in 4 mice, each one grafted with 2 different sponges. The anti-angiogenesis activity of p17 peptide was evaluated in the presence of MCFTß. The figure shows a typical experiment out of 4 different experiments that gave similar results, upon systemic administration (i.p.) of the peptide. Co-injecting p17 in the plug with MCFTß gave similar results (not shown). Dx: Doxycycline. Lower part: CD31 immunohistochemistry of sponges in the same experimental conditions shown in the upper part. **Panel C**. On the left: haemoglobin contents ± SD versus MCFM +Dx taken as 100% in explanted specimes from 4 different experiments. On the right: % vascularization ± SD in the same conditions. * p<0.05 (Student t-test), significantly different versus the relevant control.

MCFM and MCFTß-dependent angiogenesis was evaluated *in vitro* by capillary morphogenesis and *in vivo* by matrigel sponge assay. MVECs were cultivated in CM of MCFM or MCFTß, in the presence or absence of doxycycline (figure 2, panel A). On the basis of our previous data of TGFß angiogenesis on MVECs [17], we diluted CM of MCFTß to obtain a TGFß concentration of 1 ng/ml, the most efficient in stimulating an angiogenic response *in vitro*. The same concentration was used throughout the work in *in vitro* experiments, and CM of MCFM was diluted accordingly. MCFTß CM obtained in the absence of doxycycline (Dx−), produced a marked increase in capillary-like networks formation already at 6 hours after treatment, with respect to media from MCFM. Differences were maintained also after 18 h (not shown). CM from cells treated with doxycycline (Dx+) were less efficient in capillary morphogenesis promotion. Overall, these data indicate that TGFß overproduction by MCF7 cells is associated with an increase of capillary morphogenesis and that MCF7 cells used in this study are not endowed with a relevant spontaneous pro-angiogenesis activity. MVEC cells were exposed to MCFTß CM for 6–18 hours after plating on Matrigel in the presence of p17, p144 or a scramble peptide as a negative control (figure 2, bottom pictures of panel A). According to its higher solubility in aqueous solution with respect to P144 [17], the p17 inhibitor showed enhanced antiangiogenic activity. The ability of TGFß to induce angiogenesis *in vivo* was evaluated in SCID mice by Matrigel sponge assay (figure 2, panel B). A mix of Matrigel, MCF-7 transiently-transfected cells and heparin was injected subcutaneously into the flanks of SCID mice. In the absence of doxycycline (Dx−), mice injected with MCFTß showed a marked increase in angiogenesis with respect to mice injected with MCFM. The angiogenesis increase was almost completely abolished by addition of doxycycline in the injection mix (Dx+) (not shown), or in animals pre-treated with i.p. injection of doxycycline for two days before the Matrigel sponge and treated with doxycycline for the whole duration of the experiment (shown in figure 2B). In view of its high solubility, only the p17 decoy peptide was used in the Matrigel sponge assay in SCID mice. p17 administration inhibited MCFTß-dependent angiogenesis independently of the administration protocol (addition to the injection mix or injected i.p.). Angiogenesis under the described conditions was quantified by measuring the haemoglobin content in Matrigel extracts (histogram on the left of figure 2, panel C) and by counting vessels within the plug by immunohistochemistry using anti-CD31 antibodies to identify endothelial cells (figure 2, panel B and histogram on the right of figure 2, panel C).

### Canonical TGFß signaling is activated in angiogenesis promoted by TGFß-overexpressing cells

**Figure 3:**
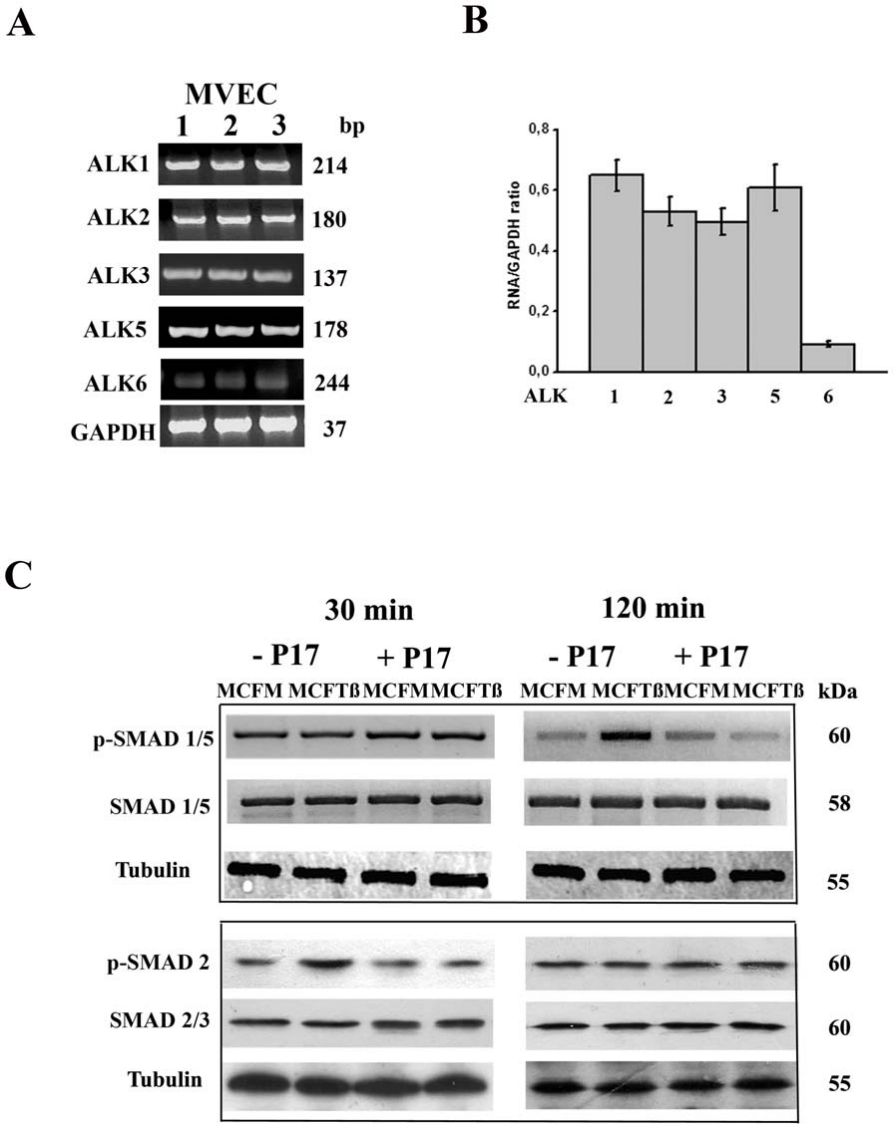
Evaluation of ALK pattern and SMAD1/2/3/5 phosphorylation in MVECs. **Panel A**. RT-PCR showing ALK expression on 3 of the MVEC cell lines used. GAPDH: loading control. Bp is shown on the right. The picture shows the results of a typical experiment out of 3 replicas. **Panel B.** Quantification of ALK RNA as related to GAPDH. The histogram shows the results obtained in 3 different experiments performed in duplicate on each MVEC cell line, ± SD. * p<0.05 (Student t-test), significantly different among various ALK RNAs. **Panel C**. Western blots of total protein extracts from MVECs exposed to CM of MCFM and MCFTß for 30 min and 120 min, respectively, in the presence or absence of P17. Western blots were probed for phospho-SMAD1/5, SMAD5, phospho-SMAD2, SMAD2/3 and Tubulin (loading control) as indicated. Molecular weights are reported on the right. Also in this case the picture shows the results of a typical experiment out of 3 replicas.

**Figure 4:**
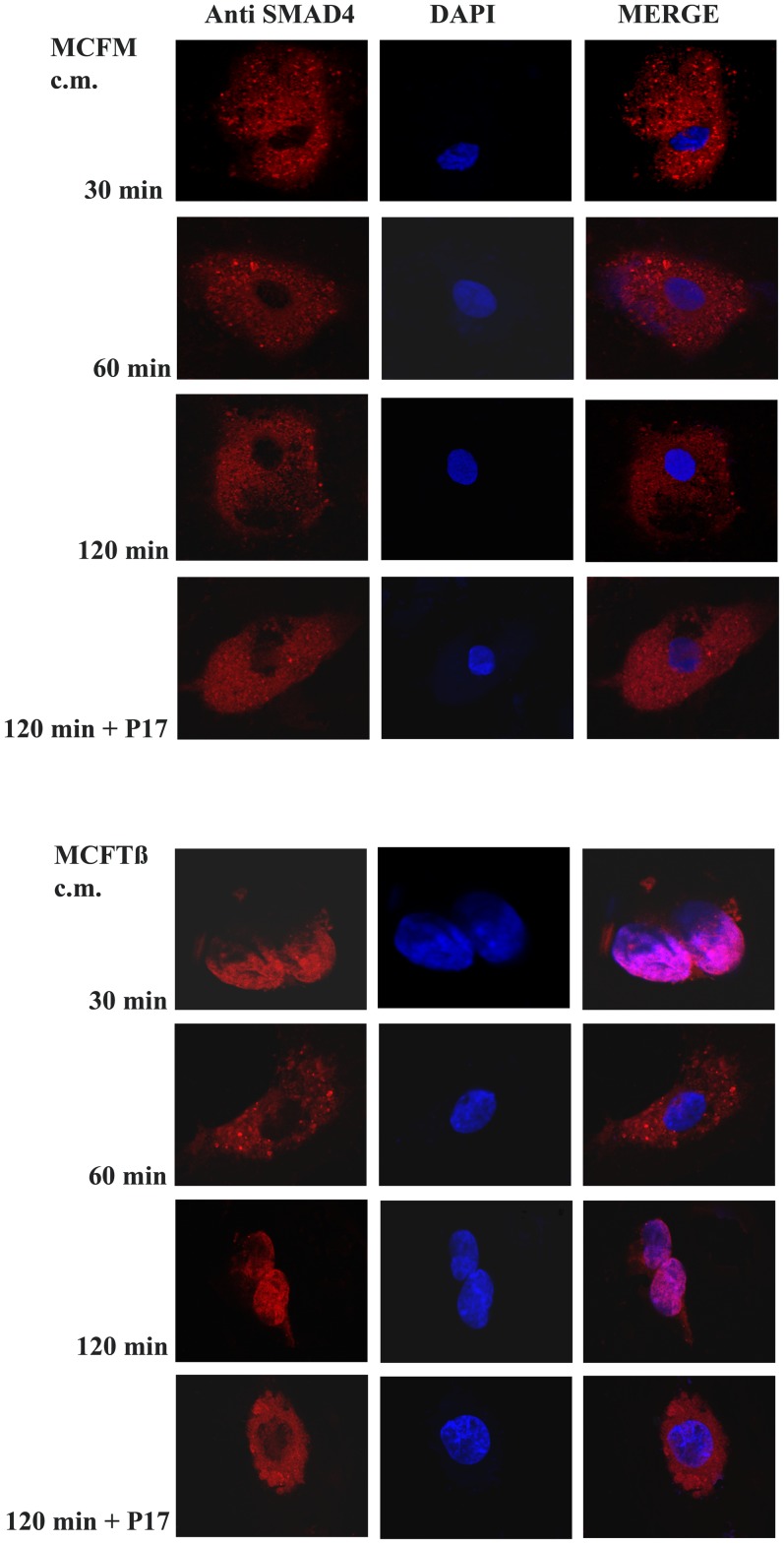
SMAD4 nuclear translocation. Numbers on the left indicate the elapsed time (hours) after addition of MCFM and MCFTß CM to MVEC. +P17: results obtained in the presence of the TGFß inhibiting peptide p17. The data show a typical experiment out of 3 replicas that gave similar results.

There are indications that EC from various sources may greatly differ with regard to the expression pattern of the ALK type I receptor sub-unit for the TGFß family ligands [44]. Therefore, we have characterized by a semi-quantitative RT-PCR the ALK expression of MVEC used in this study and have obtained the data shown in figures 3A and 3B, an ALK pattern that may support stimulation by almost all the TGFß family members. Activation of the canonical EC TGFß signaling was investigated by assessing the phosphorylation of SMAD1/5 and SMAD2/3 proteins, as well as SMAD4 nuclear translocation. Aliquots of total protein extracts from MVEC cells exposed for 2.5 hours to CM of cancer cells were analyzed by Western blot probed for p-SMAD1/5 and p-SMAD2 (figure 3C). While phosphorylation of SMAD2 was evident at 30 min following addition of MCFTß CM, the amount of phosphorylated SMAD1/5 occurred at 2 h after stimulation. p-SMAD2 returned to basal levels 90 min after stimulation, while p-SMAD1/5 appeared at 90 min following CM challenge (not shown). Treatment with anti-TGFß p17 reduced the level of both p-SMAD1/5 and p-SMAD2. It is known that, following phosphorylation, SMAD2/3 and/or SMAD1/5 bind SMAD4, giving origin to a complex which undergoes nuclear translocation and triggers transcription of a series of genes relevant for TGFß activity. Therefore, we investigated by confocal microscopy the time course of SMAD4 subcellular distribution in MVECs challenged with CM of MCFM and MCFTß (figure 4). A nuclear pattern of SMAD4 became evident 30 minutes after stimulation only with MCFTß CM, concomitant with SMAD2 phosphorylation. Such a pattern was absent in each condition 60 minutes after stimulation, but nuclear localization of SMAD4 became again evident after 2 hours, concomitant with SMAD1/5 phosphorylation, only in MVECs treated with MCFTß CM. As expected, treatment with TGFß inhibitor p17 prevented SMAD4 nuclear translocation.

### TGFß pathways are activated in endothelial cells challenged with conditioned-medium of TGFß-overexpressing cells and are controlled by anti-TGFß peptides

**Figure 5:**
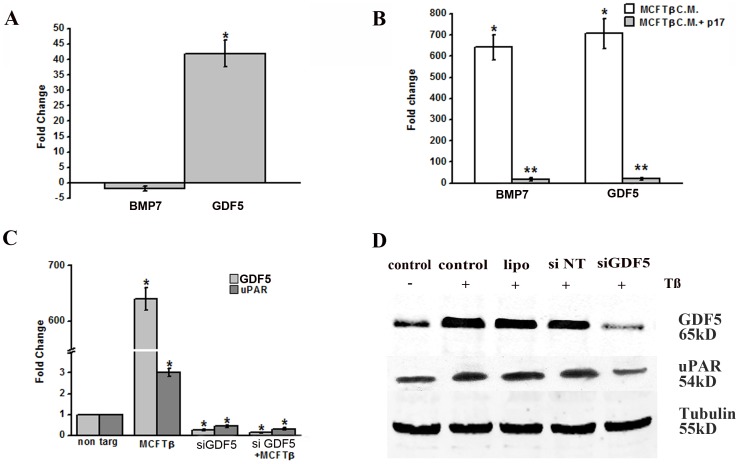
TGFß-dependent gene activation in MVECs. **Panel A**. Activation of pro-angiogenic genes following 6 h MVEC stimulation with exogenously added recombinant TGFß (1 ng/ml). BMP7, bone morphogenetic protein-7; GDF5, growth and differentiation factor-5. Data shown were obtained in 3 different experiments performed in double, and are expressed as average value ±SD. * p<0.05 (Student t-test). **Panel B**. Activation of pro-angiogenic genes following 6 h MVEC stimulation with CM of MCFM and MCFTß cells in the absence and in the presence of peptide p17. White columns: fold change of gene expression under stimulation with Dx−/MCFTß-CM with respect to MCFM-CM, assumed as value 1. Grey columns: the same of white columns, in the presence of p17. Data shown were obtained in 3 different experiments performed in double, and are expressed as average value ±SD. * p<0.05 (Student t-test) with respect to values obtained with MCFM CM; ** p<0.05 in the presence of the inhibiting peptide with respect to stimulation by Dx−/MCFTß-CM. **Panel C**. Quantitative PCR of GDF5 and uPAR in control MVECs and in MVECs stimulated with 1 ng/ml TGFß. non targ: not-targeting siRNA; siGDF5, siRNA anti-GDF5. * p<0.05 (Student t-test) with respect to values obtained with not-targeting siRNA. **Panel D.** Western blotting of MVEC lysates (100 µg/lane), under control conditions (control) and following stimulation with 1 ng/ml TGFß alone (TGFß), TGFß+lipofectamine (lipo), TGFß+not targeting siRNA (siNT) and TGFß+GDF5-targeting siRNA (siDGF5). The primary antibodies were anti-GDF5 IgG and M2 polyclonal anti-uPAR antibody. Tubulin, loading control. Molecular weights are reported on the right.

**Figure 6:**
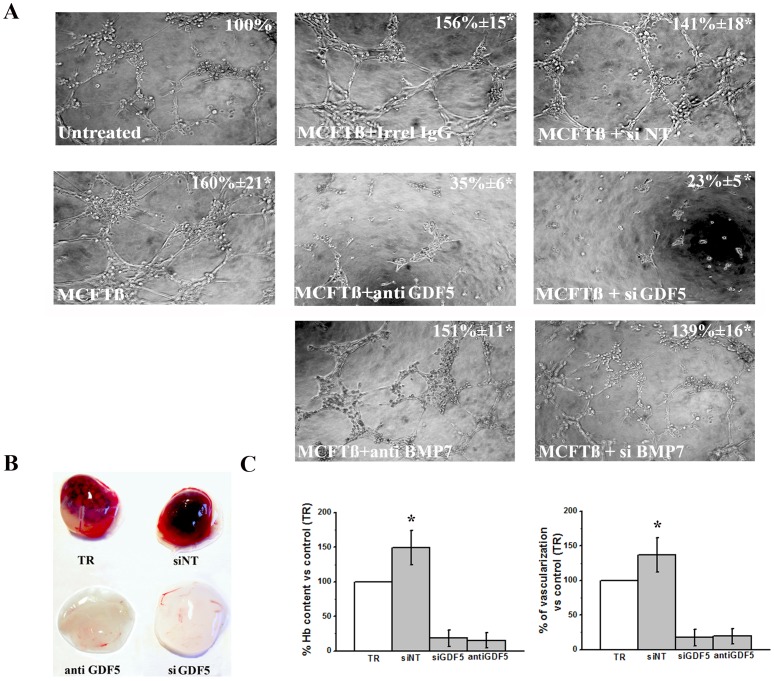
TGFß/GDF5-dependent angiogenesis in vitro and in vivo. **Panel A**. Capillary morphogenesis experiments in control MVECs (untreated), upon MCFTß CM stimulation alone, and in the presence of irrelevant IgG (+ Irrel IgG), 5 µg/ml anti-GDF5 antibody (anti-GDF5), not-targeting siRNA (siNT), GDF5-targeting siRNA (siGDF5), 5 µg/ml anti-BM7 antibody (anti BMP7), BMP7-targeting siRNA (siBMP7). **Panel B**. Representative experiment of siGDF5-dependent and anti-GDF5 antibody-dependent inhibition of angiogenesis in the Matrigel sponge model in mice. **Panel C**. On the left: angiogenesis in the sponges was quantified by evaluating the haemoglobin content of each sponge. On the right: % vascularization ± SD in the same conditions. Images were acquired with the ZEISS SR Stemi stereomicroscope. TR: transfection reagents alone; siNT: not-targeting siRNA. Results are the mean of three experiments (three animal for each condition, two Matrigel sponges in each animal). Graphs are shown as mean ± SD; * p<0.05 (Student t-test) with respect to both transfection reagents alone (TR) and not-targeting siRNA (siNT).

In order to study the pattern of gene activation expressed by MVECs treated with medium of conditionally transfected MCF7, a qPCR based assay was used for quantitative profiling of a set of 94 preselected genes related to TGFß pathways. Besides direct challenge of MVEC with recombinant TGFß (1 ng/ml), three different experiments (MVECs stimulation with CM from MCFM and MCFTß, in the absence and in the presence of p17 peptide inhibitor) were performed. In order to derive biological meaning from the differentially regulated genes, we performed the functional classification of all the involved genes on the basis of information from the Gene Ontology database (Molecular Function, Biological Process and Cellular Component) and from data collected in the scientific literature. Only genes involved in the angiogenesis process were selected. Figure 5A shows that, at 6 hours, stimulation of MVECs with exogenously added recombinant TGFß induced a more than forty fold over-expression of the Growth and Differentiation Factor-5 (GDF5), a pro-angiogenic BMP [45]. When MVECs were stimulated for 6 hours with CM from MCFM and MCFTß, in the absence and in the presence of p17 peptide inhibitor (figure 5B), we observed a striking up-regulation of both GDF5 and of BMP7, another BMP whose co-operative role in TGFß angiogenesis has been emphasized [46]. The p17 peptide completely reverted over-expression of each relevant molecule, showing that expression of up-regulated BMPs is dependent on TGFß stimulation of MVECs. Figure 5C shows a quantitative PCR indicating that: a) stimulation of MVECs with MCFTß CM up-regulates both GDF5 and uPAR expression; b) an anti-GDF5 siRNA down-regulates both GDF5 and uPAR expression either in the absence or in the presence of MCFTß CM stimulation. Stimulation with recombinant TGFß gave similar results (not shown). Figure 5D shows the Western blotting with anti-GDF5 IgG (5 µg/ml) and anti-uPAR M2 polyclonal antibody (5 µg/ml) of aliquots (100 µg) of MVEC lysates under control conditions, and after stimulation with MCFTß CM, in the absence (control) or in the presence of lipofectamine alone, of lipofectamine containing not-targeting RNA (siNT, not-targeting) and of lipofectamine containing anti-GDF5 siRNA. The results shown in figure 5C show that uPAR up-regulation, previously shown to drive TGFß-dependent angiogenesis [17], is regulated by TGFß-dependent GDF5 up-regulation. Figure 6A shows capillary morphogenesis experiments performed with untreated MVECs (taken as 100%), and with MVECs challenged by MCFTß CM, in the presence of irrelevant IgG, of anti-GDF5 IgG, of siNT (not-targeting) and of siGDF5. Both anti-GDF5 treatments inhibited MCFTß CM-induced capillary morphogenesis. Similar results (not shown) were obtained in capillary morphogenesis experiments upon stimulation with exogenously added recombinant TGFß. Interestingly, antibodies and siRNA targeting BMP7 did not inhibit Tet-Off conditioned-media-dependent capillary morphogenesis of MVEC (figure 6A), which implies GDF5 as the only BMP positively involved in TGFß-dependent angiogenesis and uPAR up-regulation in MVECs. Taken together, these data strongly indicate that TGFß-dependent MCF7 angiogenesis depends on induction of GDF5 in MVECs, which, in turn, up-regulates uPAR expression. On these basis we have performed *in vivo* Matrigel sponge assays (figure 6B), co-injecting in C57/BL6 male mice Matrigel containing CM of MCFTß cells obtained after doxycycline subtraction, with or without GDF5-siRNA, combined with DharmaFECT, siNT combined with DharmaFECT, or DharmaFECT alone (transfection reagent, TR). Also anti-GDF5 antibodies were used to block the GDF5 effects (same figure) . Results clearly show that, even in this *in vivo* setting, silencing or inhibition of GDF5 inhibits TGFß-dependent MCF7 angiogenesis.

## Discussion

In this paper we have shown that TGFß produced by breast cancer cells induces in endothelial cells GDF5 expression, which in turn stimulates angiogenesis both *in vitro* and *in vivo*. Angiogenesis activation is very rapid and the involved mechanism is totally opposed to the old and controversial dogma about the AKL5/ALK1 balance. We have inserted in human MCF7 breast cancer cell line a mutated TGFß gene in a tetracycline-repressible vector in order to obtain conditional expression of mature TGFß in the absence of tetracycline upon transient transfection. Transfected MCF7 cells expressed several characteristics of the EMT and did not show viability decrease or caspase-3 activation. The conditioned-medium of TGFß-over-expressing MCF7 cells (MCFTß) stimulated angiogenesis *in vitro* and *in vivo*, which was inhibited by TGFß inhibitor peptides upon local or systemic administration. We have also shown early activation of TGFß SMAD2/3, followed by activation of SMAD1/5 signaling in MVEC by MCFTß cells, along with SMAD4 nuclear translocation and transcription of pro-angiogenic molecules, including uPAR and GDF5. All these events underwent anti-TGFß peptides-dependent inhibition. Thus MCF7 overproduction of TGFß and the subsequent chain of pro-angiogenic effects may be controlled by an efficient TGFß antagonization. Our data, showing phosphorylation of both SMAD2/3 and SMAD1/5 in MVECs upon stimulation with TGFß, are in agreement with the reported TGFß transduction pathways in EC [14], [46]. The analysis of a set of genes related to TGFß pathway has allowed to show for the first time that the most relevant changes of gene expression induced in MVEC by breast carcinoma cells conditionally induced to over-express TGFß, included some members of the BMPs family. BMPs are intercellular signaling molecules with multiple functions during development and differentiation [47], [48]. BMPs belong to the TGFß superfamily of growth factors and bind to heteromeric transmembrane serine/threonine kinase receptors [48]–[51]. Such interaction triggers downstream signalling cascades that are strictly dependent on the sub-family of the involved BMPs and that involve angiogenesis for several BMPs [23], [29], [51]. Angiogenesis is a multistep process that can be divided in two phases. During the initial “activation” phase, the perivascular basement membrane is degraded, endothelial cells migrate in the extracellular space, proliferate, form capillary sprouts and organize in tubular-like structures. In the subsequent “maturation-stabilization” phase EC stop invasion and proliferation, the basement membrane is rebuilt and smooth muscle cells/pericytes are recruited in order to surround neo-vessels, thus reconstituting vessel wall integrity. Recent observations clearly emphasize the emerging role of BMPs in angiogenesis [23], [52], leading to the proposal of a BMPs balance which controls angiogenesis activation and maturation [52]. The proposed model distinguishes among the BMP2/4, the BMP5/6/7 and the GDF5/6/7 subgroups (which controls the activation phase of angiogenesis) from the BMP9/10 subgroup (which regulates only the maturation phase). Among the angiogenesis-related BMPs, only BMP9/10 signalling involves the ALK1 pathway, while all the other subfamilies signal through ALK2/3/6. In all the cases, pathway activation involves SMAD1/5/8 phosphorylation and SMAD4 nuclear translocation, which regulates transcription of target genes involved with angiogenesis activation/maturation. Endoglin and betaglycan are co-receptors, expressed in EC, which regulate the availability of BMPs to type I and type II receptors. Here we have shown that direct stimulation of MVEC by MCFTß CM and exogenous TGFß is characterized by a strong over-production of GDF5, which up-regulates uPAR in MVEC and stimulates the formation of capillary networks *in vitro*. These data are in agreement with our previous results showing that uPAR up-regulation is mandatory in TGFß-dependent angiogenesis [17]. MVEC used in this work express the type-I receptors ALK1/2/3/5/6 and are therefore available to interact with every member of the TGFß/BMP superfamily, including the ALK2/3/6-related GDF5. GDF5 silencing impaired MCFTß-CM-dependent angiogenesis *in vitro* and *in vivo*. GDF5 was the first BMP reported to have a role in angiogenesis. GDF5 enhances angiogenesis in the chorio-allantoic membrane (CAM) assay and in the rabbit cornea assay [45], by increasing aortic EC migration but not their proliferation. On the other side, in the context of conditional TGFß over-production by MCF7 cells, we have observed that MVEC stimulation results into overproduction not only of GDF5, but also of BMP7. Both these molecules have been recognized to be implicated in angiogenesis [45], [46]. However, validation of our microarray data obtained by Western blotting, specific antibodies-inhibition, siRNA and capillary morphogenesis, point to GDF5 as the only BMP involved in MCF7-mediated TGFß-dependent angiogenesis in our model system. At present we are unable to explain why only GDF5 is involved in angiogenesis stimulation. Since BMP7 has been shown to be pro-angiogenic in synergy with TGFß but only at very high concentrations of both molecules [46], we hypothesize that the concentrations obtained under our experimental conditions do not reach the threshold levels required to synergize in angiogenesis promotion. Recent papers have clearly shown that genetic and pharmacological targeting of ALK1 impairs tumor angiogenesis and growth by impairment of BMP9 signalling [32], [53]. On the basis of our data on GDF5-mediated TGFß induction of angiogenesis, it is now possible to suggest a putative series of events triggered by the initial interaction of TGFß with its receptors and culminating with a GDF5-dependent activation of angiogenesis. Our data, albeit obtained in an artificial TGFß-dependent angiogenesis tumor model, provide the first description of an activation of these events in EC as an effect of conditional TGFß-overexpression in cancer cells. The evidence that anti-TGFß peptides treatment efficiently blocks TGFß angiogenesis even upon systemic administration, renders these molecules particularly interesting for a possible pharmacological control of mammary cancer TGFß-switch. However, it is common experience that peptides so far have not done well in clinical translation. Antibody targeting has much better characteristics in vivo and in patients than do most peptides, suggesting this could be an interesting approach. Targeting GDF5 may also leave intact some of the anti-tumor activities of TGFß, while blocking pro-tumor activities.

## Conclusions

Conditional TGFß production by MCF7 breast cancer cells induces angiogenesis both *in vitro* and *in vivo*. In our model, angiogenesis activation depends on TGFß-dependent production of growth and differentiation factor-5 (GDF5) in target endothelial cells, a mechanism that is different from the old and controversial dogma about the AKL5/ALK1 balance. The GDF5-dependent pro-angiogenic effects of TGFß are controlled by anti-TGFß peptides and anti-GDF5 antibodies. These preclinical data provide a basis to develop researches aimed to validate our observations in breast tumors with the aim to use anti TGFß–peptides and anti-GDF5 antibodies as possible therapeutic tools for TGFß/GDF5–dependent breast cancer angiogenesis.
